# Structural Brain Correlates of Poor Reading Comprehension

**DOI:** 10.1162/NOL.a.265

**Published:** 2026-07-01

**Authors:** Kelly Mahaffy, Nabin Koirala, Daniel Kleinman, Nicole Landi

**Affiliations:** Department of Psychological Sciences, University of Connecticut, Storrs, CT, USA; Child Study Center, Yale School of Medicine, New Haven, CT, USA; Brain Imaging Research Core, University of Connecticut, Storrs, CT, USA; The Nathan S. Klein Institute for Psychiatric Research, Orangeburg, NY, USA

**Keywords:** educational neuroscience, NODDI (neurite orientation dispersion and density imaging), poor comprehension, reading comprehension

## Abstract

Poor comprehenders (PCs) have typical word reading skill and intelligence but poorer than expected reading comprehension. While the prevalence of PCs is similar to that of poor decoders (individuals who struggle fluently converting written text into spoken language), less is known about the neurobiological substrates of poor comprehension. Extant studies have found small differences in gray matter volume between PCs and poor or typically reading peers. However, a detailed quantification of cortical morphometric features and white matter integrity remains unexplored. Data from 2,100 children (1,200 with imaging data), aged 8–16 years were analyzed to determine if there is a distinct neuroanatomy associated with poor reading comprehension across three common methods for classifying PCs. We computed gray matter volume, cortical thickness, and surface area, as well as white matter measures (mean diffusivity, fractional anisotropy, neurite orientation, and neurite density) for PCs and compared these measures with those of poor decoders and typical readers. Results revealed small but widespread white matter differences, but no gray matter differences between PCs and other readers. PCs showed decreased white matter integrity (increased mean diffusivity, decreased neurite density) in tracts previously associated with reading, including the superior longitudinal fasciculus and inferior longitudinal fasciculus, and in tracts that have been associated with cognitive performance such as the uncinate fasciculus. These results suggest that diffuse structural connectivity differences may underlie reading comprehension weaknesses in the face of intact decoding skills. This is consistent with the behavioral profile of PCs who exhibit a broad pattern of subclinical impairments in language and integrative cognitive processes.

## INTRODUCTION

Reading is vital for both academic and personal success, yet there is substantial variation in reading comprehension with recent reports documenting poorer than expected reading comprehension performance among both children and adults in the United States and beyond (Gough & Tunmer, 1986; Guo et al., 2022; Kim, 2017, 2020; NAEP, 2009; Perfetti & Adlof, 2012; Perfetti & Stafura, 2014; van den Broek et al., 1999). Neurobiological studies of reading have been instrumental in documenting individual differences in single-word reading and decoding, which has helped to isolate possible mechanisms of reading variation (Aboud et al., 2016; Arrington et al., 2017; Cutting et al., 2013; Eckert et al., 2017; Feng et al., 2023; Horowitz-Kraus et al., 2014, 2015; Koirala et al., 2021; Murphy et al., 2019; Patael et al., 2018; Paulesu et al., 2014; Richlan, 2012, 2014; Ryherd et al., 2018; Van Der Auwera et al., 2021). However, there are far fewer neurobiological studies of reading comprehension and individual differences therein. A small body of literature documents functional and structural differences between good comprehenders and poor comprehenders (PCs) as elaborated below; however, this literature is plagued by small sample sizes, variable classification of PCs, and inconsistent findings. The purpose of the current study was to provide the first large-scale investigation of structural neuroanatomic characteristics of good comprehenders and PCs as compared to poor decoding and typically reading peers.

**Figure 1.**
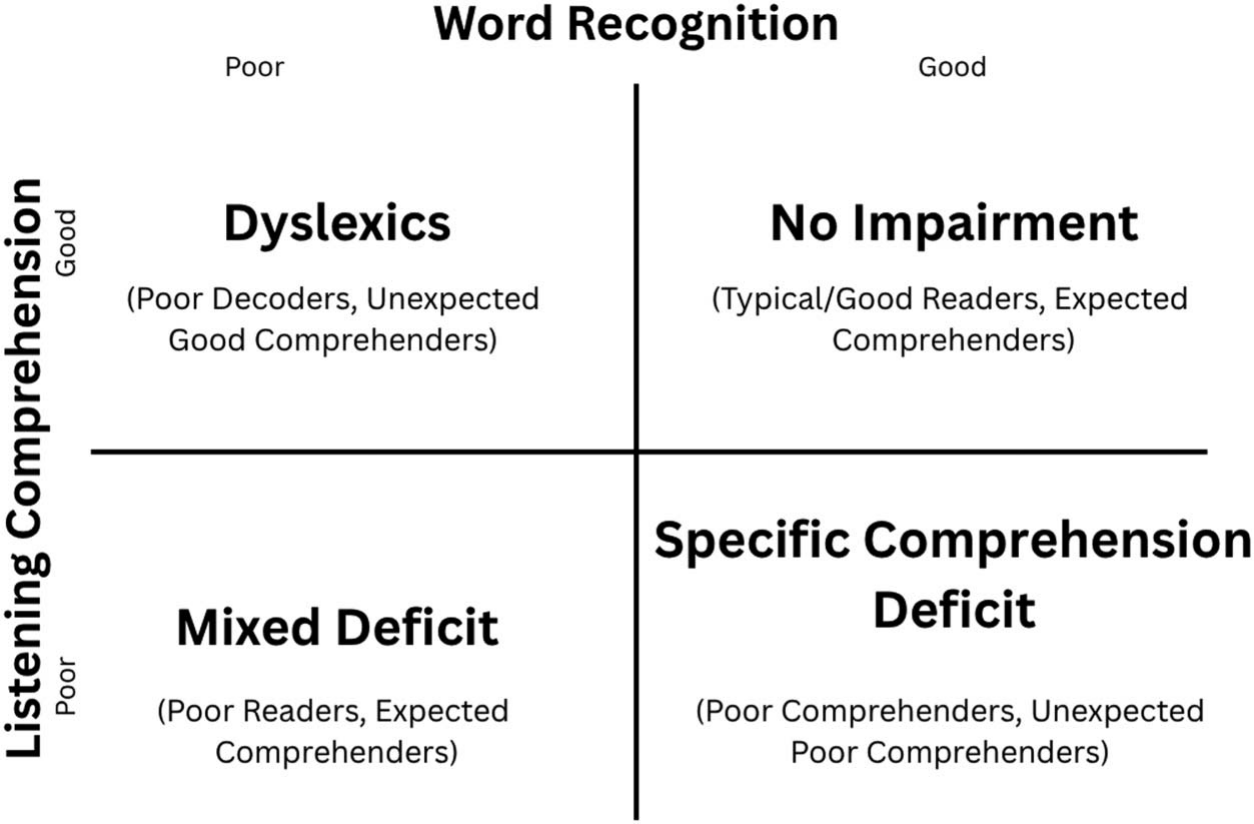
A visual schematic of the Simple View of Reading and related quadrant models, originally proposed by Gough and Tunmer (1986) and visually depicted by Catts et al. (2006), depicting how variation in listing comprehension and/or word recognition (decoding) can influence individual differences in reading and lead to different types of poor readers. Rough mappings for how these categories map onto the groups in our cutoff classification and mixed regression analysis are also included in parentheses.

It is well understood that individuals can vary in their performance across multiple levels of reading skill, from single-word decoding to text comprehension (Catts et al., 2003; Gough & Tunmer, 1986; Hoover, 2024; Landi, 2010). This variation is well described by the Simple View of Reading, which delineates reading comprehension as the product of decoding and listening comprehension, such that neither one alone is sufficient, and variation in either produces variation in reading comprehension (Gough & Tunmer, 1986). Related quadrant models characterize and identify four groups of readers as a function of their listening comprehension and decoding skills (see Figure 1; Catts et al., 2003; Gough & Tunmer, 1986; Hoover, 2024; Landi, 2010). While substantial research has focused on poor decoders/dyslexics and generally poor readers, relatively less work has focused on individuals with good decoding skills but poor reading comprehension, so called “PCs” (Barquero & Cutting, 2021; Peterson & Pennington, 2012; Snowling et al., 2020).

In addition to poor reading comprehension relative to decoding, these readers tend to have subclinical weaknesses in oral language skills, including smaller vocabularies and weaker morphological and grammatical processing, but no diagnosis of a clinical or learning disability (Adlof & Catts, 2015; Catts et al., 2006; Colenbrander et al., 2016; Spencer et al., 2019; Tong et al., 2011, 2014). PCs also tend to have poorer than average performance on complex integrative tasks, such as inference making and comprehension monitoring (Cain & Oakhill, 1999; Catts et al., 2006; Kim, 2017; Yang et al., 2005) as well as subtle processing weaknesses in domain-general skills such as executive functions and category learning (Bailey et al., 2016; Barquero & Cutting, 2021; Cirino et al., 2019; Ryherd & Landi, 2019; Wagner et al., 2021). Although PCs are now an accepted group of poor readers, there is debate as to whether they are best characterized as having subclinical developmental language disorder (DLD) or under a multifactorial model with both language and more domain-general cognitive difficulties that may stem from independent causal mechanisms (Barquero & Cutting, 2021; Earle & Del Tufo, 2021; James et al., 2023; Landi & Ryherd, 2017; Nation & Norbury, 2005; Spencer et al., 2020; Wagner et al., 2021). Moreover, rather than discrete groups, recent large studies reveal a continuous distribution of reading comprehension relative to word decoding skill with discrepancy present at all skill levels (Guo et al., 2022; Patael et al., 2018; Spencer et al., 2019, 2020; Wagner et al., 2021). While neurobiological studies could help to shed light on these issues, at present there are only a few studies that have examined brain structure or function in PCs. These studies tend to have small samples, and variation in the methods used across studies limits generalizable conclusions. Below, we review the small set of neuroimaging studies on PCs and the motivation behind the current study.

Two previous fMRI studies used matched group designs to compare functional brain activation in PCs to that of typical readers and/or poor decoders. The first study (Cutting et al., 2013) found increased activation for PCs (*n* = 12 adolescents, ages 10–14 years) as compared to poor decoders (*n* = 20 adolescents, ages 10–14) during single-word reading (lexical decision) in the left fusiform gyrus, left middle frontal gyrus, and left cingulate gyrus. These findings are consistent with the better single-word reading performance that is typically observed in PCs relative to dyslexic readers (Cutting et al., 2013). When compared to typical readers (*n* = 19 adolescents, ages 10–14 years), PCs had less activation in the right lingual gyrus and left cuneus for low-frequency words only and greater activation in the left inferior frontal gyrus (IFG) compared to the right IFG. These findings are consistent with differential access to semantic information from visual information between PCs and typical readers. Finally, connectivity analyses revealed that PCs had greater functional connectivity relative to typical readers between the left IFG and a number of cortical and subcortical regions including the left parahippocampal and hippocampal gyri and right thalamus, putamen, and middle frontal gyrus, suggesting less efficient or less automatic access of lexical semantic information in PCs relative to typical readers (Cutting et al., 2013).

A second study (Feng et al., 2023) used an auditory rhyme judgment task and found reduced activation for PCs (*n* = 16 fifth-graders) relative to typical readers (*n* = 16 age-matched controls) in the left middle frontal gyrus, IFG, insula, inferior temporal gyrus/middle occipital gyrus (fusiform gyrus), and anterior cingulate gyrus. Increased activation was observed in the right precentral, anterior cingulate, and supramarginal gyri and in the left posterior cingulate, angular gyrus, middle frontal gyrus, and medial superior frontal gyrus (Feng et al., 2023). The differences observed here with Chinese speakers, which implicate both language/reading associated regions and more domain general regions, stand in partial contrast to previous behavioral findings, which reveal typical rhyme processing for PCs (Landi & Perfetti, 2007). However, note that the PCs in this study had significantly poorer character naming than typical readers and thus may not be comparable to most studies of PCs, wherein decoding skills are matched (Feng et al., 2023). Moreover, psycholinguistic differences between English and Chinese, including the use of tones to distinguish meaning in Chinese and the greater opacity of the Chinese writing system, complicate direct comparisons.

Two additional fMRI studies have investigated brain activation associated with reading comprehension skill during passage reading and/or listening using regression-based approaches, which control for decoding skill and general cognitive skill. For example, Aboud et al. (2016) used a passage reading task with 38 adolescents who were 9–14 years old and found that those with better reading comprehension showed increased activation in the left IFG extending into the left insula and dorsolateral prefrontal cortex (dlPFC). Additional positive correlations were found between reading comprehension and bilateral temporoparietal activation extending into the right ventral IFG, ventral insula, and left IFG as well as the default mode network (Aboud et al., 2016). These findings might suggest enhanced syntactic and/or semantic processing, narrative structure building, and comprehension monitoring for better comprehenders. Using a similar approach, Ryherd et al. (2018) examined functional activation during alternative blocks of passage reading and story listening in 32 adolescents ages 13–18 years. Similar to Aboud et al., they found that those with better reading comprehension had increased activation across a broad network of regions important for language, including the left IFG, fusiform gyrus, middle temporal gyrus, and left cerebellum during both reading and listening. They also observed that those with lower reading comprehension had greater activation in regions linked to attention and executive functions, including bilateral anterior and posterior cingulate, right middle frontal gyrus, and right cerebellum, which could suggest more effortful processing in this paradigm (Ryherd et al., 2018).

Although the functional tasks, languages/writing systems, and the methods used to identify the neural signatures of comprehension differed across these studies, neural findings tended to mirror reader characteristics across studies. When comprehension was sufficiently isolated from decoding, greater activation for better comprehenders was observed in lexical semantic processing regions during lexical decision and in higher level language regions and/or regions thought to be involved in comprehension monitoring or retrieval during text processing or listening comprehension. Some of these studies also observed greater activation for PCs, typically in regions associated with executive functions, though also in some regions linked to higher level language processing. Importantly, differences between PCs and their peers were not confined to the left hemisphere: Most studies reported effects in both hemispheres, with substantial right-hemisphere involvement, including regions implicated in integrative language processing and executive control. Given that these studies focused on adolescents and adults, this overall pattern of findings likely represents a combination of atypical pathways for language and/or executive functions and compensation for weaknesses in the primary language processing systems. However, study differences in task, population, PC classification approach, and small samples preclude strong conclusions.

With respect to brain structure, two studies have investigated gray matter volume in PCs relative to typical readers and poor decoders. Using multivariate pattern analyses and a matched group design, Bailey et al. (2016) analyzed data from 41 adolescents and found reduced gray matter volume for PCs (*n* = 11, mean age = 11.5 years) relative to poor decoders (*n* = 14, mean age = 12.5 years) and typical readers (*n* = 16, mean age = 11.9 years) in a network of bilateral regions that subserve language and more general cognitive functions. Regions identified included the following: right hemisphere: superior/middle frontal gyrus, superior/inferior temporal gyrus, precentral gyrus, anterior cingulate, and the cuneus and cerebellum; and left hemisphere: superior frontal gyrus, inferior/superior temporal gyrus, and middle occipital gyrus. No regions were found to be significantly larger for PCs relative to poor decoders and typical readers (Bailey et al., 2016). While this study identified a relatively broad network of structural differences, many of the identified regions overlap with those where functional activation was greater for better comprehenders (controlling for decoding) during text processing, suggesting some continuity across structure and function. A second study of gray matter volume by Patael et al. (2018) used a regression-based approach with 55 participants ages 10–16 years old to attempt to identify regions associated with a discrepancy between reading comprehension and decoding. They found that gray matter volume in the left dlPFC was related to reading comprehension and the discrepancy between reading comprehension and decoding performance, such that participants with higher reading comprehension performance relative to their decoding performance (unexpected good comprehenders) had increased gray matter volume in the left dlPFC compared with those with relatively higher decoding than reading comprehension (Patael et al., 2018). Notably, activation in a similar region (left IFG into left dlPFC) was associated with better reading comprehension (controlling for decoding) during text reading in similarly aged children in the Aboud et al. and Ryherd et al. studies discussed above.

While there are no studies of PCs that we know of that have examined white matter, given inclusion of white matter in the current study, we note two studies that have looked at correlations between reading comprehension and white matter integrity (without controlling for decoding or word-level reading) in 21 young adults (ages 15–19 years) and 23 children (mean age = 8.5 years), respectively. Both studies found a positive correlation between reading comprehension and fractional anisotropy (FA) in the bilateral arcuate fasciculus (AF), inferior longitudinal fasciculus (ILF), and superior longitudinal fasciculus (SLF) (Horowitz-Kraus et al., 2014, 2015). While we use these studies to guide our white matter predictions in the absence of studies of PCs specifically, it is important to note that integrity in these same tracts has also been correlated with word-level reading skills, and thus, associations may not be specific to reading comprehension after controlling for decoding or word-level reading skills.

Collectively, these structural findings are partially consistent with the functional findings and suggest broad associations between reading comprehension and brain anatomy in both language-associated and more domain-general processing linked regions, across both the left and right hemispheres. However, with only two relatively small studies of gray matter structure in PCs that used different analytic approaches and no white matter studies of PCs, this conclusion is tenuous and incomplete.

To advance understanding of the neurobiological basis of PCs and, in turn, clarify potential mechanisms underlying their difficulties, the present study analyzes gray and white matter correlates of poor reading comprehension in the absence of decoding deficits. Using a large subset (*n* > 2,000) of a publicly available data set of children, this work represents the largest investigation to date, focused on the neurobiology of poor comprehension. Because no prior study has examined white matter structure in this population, incorporating white matter imaging is expected to be particularly informative. White matter is also theoretically relevant: reading comprehension relies on coordinated processing across multiple anatomically distributed regions, making the integrity of inter-regional communication pathways essential.

Additionally, given the wide range of methods previously used to disentangle reading comprehension from decoding, we applied three common classification approaches to evaluate whether the choice of method influences the brain regions or tracts identified. These included (a) a cutoff-based model that identifies PCs using standard scores reflecting poor comprehension but intact decoding; (b) a mixed classification model in which reading comprehension is predicted from decoding, vocabulary, age, sex, and nonverbal IQ, with participants categorized by residuals; and (c) a continuous approach that discards categorical grouping and uses residuals from the mixed classification model as continuous predictors of brain measures.

Drawing on converging findings from prior functional and structural neuroimaging studies of good comprehenders and PCs, we formulated the following predictions: (a) PCs/unexpected PCs would exhibit reduced gray matter morphometry in bilateral language- and attention-related regions—including the right superior and middle frontal gyri, superior and inferior temporal gyri, precentral and postcentral gyri, anterior cingulate, and lingual gyrus, as well as the left superior frontal gyrus, superior and inferior temporal gyri, middle occipital gyrus, inferior parietal lobule, dlPFC—and in the hippocampus, relative to other readers (Bailey et al., 2016; Feng et al., 2023). (b) PCs/unexpected PCs would show reduced white matter integrity (i.e., lower FA and higher mean diffusivity [MD]) bilaterally in the AF, SLF, and ILF (Arrington et al., 2017; Horowitz-Kraus et al., 2014, 2015).

## MATERIALS AND METHODS

### Participants

Behavioral data and neuroimaging scans were selected from 4,748 children, aged 5–21 years, from the Child Mind Institute Healthy Brain Network biobank (Alexander et al., 2017). All participants or their parents gave informed consent to participate in the study in accordance with the Declaration of Helsinki and the Chesapeake Institutional Review Board (Alexander et al., 2017). To be included in the current study, participants had to be at least 8 years of age, have a nonverbal IQ score of ≥70, and have data for all behavioral assessments of interest. To avoid inclusion of participants who may have had difficulty understanding assessment instructions or may have more serious neurobiological anomaly, we excluded participants with clinician-confirmed diagnosis of autism and/or intellectual disability or a related disorder (see Supporting Information Table 2; Supporting Information can be found at https://doi.org/10.1162/NOL.a.265). To maintain a sample representative of the general population, participants with other mild conditions or learning disabilities (e.g., attention-deficit/hyperactivity disorder, anxiety) were not excluded. Consistent with this, participants with diagnoses equivalent to DLD, which have a partially overlapping profile with PCs, were not excluded. While there is some inconsistency on whether and how children with DLD are included in studies of PCs, with most studies of older PCs lacking history on DLD, we decided to include them and allow our classification methods determine where they fell.^1^ The final sample included 2,129 participants (age in years, *M* = 11.11, *SD* = 2.23), 1,270 of whom had 3T T1w MRI data available, with 903 participants also having diffusion data available.

### Brain Image Acquisition and Processing

All Healthy Brain Network Biobank brain imaging data used in this analysis (whole head) were acquired on 3-T scanners using protocols detailed elsewhere (Alexander et al., 2017; O’Connor et al., 2017). T1w images were acquired with a repetition time (TR) = 2,500 ms, echo time = 3.15 ms, voxel size = 0.8 mm (isometric), and flip angle = 8°. Diffusion weighted images were obtained in 64 directions with bvals of 0, 1,000, and 2,000 s/mm^2^ and isotropic voxels of 1.8 mm. Raw images were downloaded in the standard Brain Imaging Data Structure (BIDS) format and were processed in-house.

#### MRI quality control and preprocessing of images

T1w image quality was assessed using MRIQC (Version 22.0.6) and then processed using the FreeSurfer (Version 7.1.1) automated pipeline for surface-based cortical reconstruction and volumetric segmentation (Dale et al., 1999; Esteban et al., 2017). The Freesurfer image analysis suite is documented and freely available for download online. The technical details of these procedures are described in prior work (Dale et al., 1999; Dale & Sereno, 1993; Fischl et al., 2001, 2002; Fischl & Dale, 2000; Fischl, Salat, et al., 2004; Fischl, Sereno, & Dale, 1999; Fischl, Sereno, Tootell, & Dale, 1999; Fischl, van der Kouwe, et al., 2004; Han et al., 2006; Jovicich et al., 2006; Reuter et al., 2010, 2012; Ségonne et al., 2004). Briefly, this processing includes motion correction and averaging of volumetric T1-weighted images (Reuter et al., 2010), removal of nonbrain tissue using a hybrid watershed/surface deformation procedure (Ségonne et al., 2004), automated Talairach transformation, segmentation of the subcortical white matter and deep gray matter volumetric structures (Fischl et al., 2002; Fischl, Salat, et al., 2004), intensity normalization (Sled et al., 1998), tessellation of the gray matter white matter boundary, automated topology correction (Fischl et al., 2001; Ségonne et al., 2007), and surface deformation following intensity gradients to optimally place the gray/white and gray/cerebrospinal fluid borders at the location where the greatest shift in intensity defines the transition to the other tissue class (Dale et al., 1999; Dale & Sereno, 1993; Fischl & Dale, 2000). Once the cortical models are complete, a number of deformable procedures were performed for further data processing and analysis, including surface inflation (Fischl & Dale, 2000), registration to a spherical atlas that is based on individual cortical folding patterns to match cortical geometry across subjects (Fischl, Sereno, & Dale, 1999), parcellation of the cerebral cortex into units with respect to gyral and sulcal structure (Desikan et al., 2006; Fischl, van der Kouwe, et al., 2004), and creation of a variety of surface-based data, including maps of curvature and sulcal depth. Processed data were mapped to the Destrieux atlas to extract cortical thickness, surface area, and volume, and a whole brain approach was used for all subsequent analyses, testing each of the 74 regions in each hemisphere for 1,270 participants (Dale et al., 1999).

Diffusion MRI (dMRI) images were processed using Functional Magnetic Resonance Imaging of the Brain Software Library (FMRIB FSL, Version 6.1.0) (Bastiani et al., 2019; Jenkinson et al., 2012). The Quality Assessment of All dMRI data (QUAD) toolbox in FSL was used to estimate multiple quality metrics including contrast-to-noise ratio (CNR) for all diffusion tensor imaging (DTI) data (Bastiani et al., 2019). Standard pipelines and general best practices for preprocessing diffusion data in FSL are well outlined elsewhere (Behrens et al., 2007; Jenkinson et al., 2012), and the specifics of diffusion processing for the data in this project are similarly outlined elsewhere (Koirala et al., 2018, 2021, 2022). In short, data were preprocessed for artifact correction including susceptibility, eddy current correction, and correcting for head movement. The Brain Extraction Toolkit was then used to create individual masks for each brain, isolating the brain from the skull. FSL’s diffusion tensor modeling tool, DTIfit, was used to obtain diffusion measures, including FA and MD. The crossing fibers distribution was estimated using BEDPOSTX, and the probability of major fiber directions were calculated. A multifiber model was fit at each voxel for tracing fibers through regions of crossing or complexity. XTRACT was used to compute probabilistic tractography (curvature threshold of ±80°, max streamline steps: 2,000) in each participant’s native space to obtain 23 major fiber tracts within the human brain. The normalized fiber probability distribution obtained was then thresholded and binarized using fslmaths to get a tract mask. The mask generated was inspected for accuracy, and the value was adapted accordingly to ensure correct segmentation. The obtained binary mask was then multiplied by the subject’s neurite orientation dispersion and density imaging (NODDI) and diffusion maps to get a tract-specific distribution of those values. All tracts, along with their abbreviations, are listed in Supporting Information Table 1.

Additionally, to better characterize the white matter microstructure, the NODDI model was fit using the NODDI toolbox (Zhang et al., 2012). NODDI is a multicompartment, non-Gaussian, biophysical tissue model that can quantitatively evaluate specific microstructural changes to distinguish between three microstructural environments: intracellular, extracellular, and cerebrospinal fluid (CSF) compartments (Zhang et al., 2012). The amount of diffusion in each of these individual compartments allows for the estimation of density and orientation distribution of axons and dendrites, collectively termed “neurites.” NODDI coefficients neurite orientation and dispersion index (ODI) and neurite density index (NDI) were mapped along 23 fiber tracts, and mean values for each tract were used for all subsequent analyses.

### Behavioral Measures

To categorize children into PC, poor decoder, or typical reader groups, behavioral assessments of reading and language were used. Reading comprehension was measured through the Wechsler Individual Achievement Test–Third Edition (WIAT-3) reading comprehension subtest, during which participants read short expository and narrative passages and answer comprehension probe questions (Pearson, 2009). Decoding was measured using the Test of Word Reading Efficiency–Second Edition pseudoword decoding efficiency subtest, during which participants read as many pronounceable English pseudowords as they are able in 45 s (Torgesen et al., 2012). Scores reflect the number of pseudowords that are correctly pronounced based on English grapheme–phoneme rules. Receptive vocabulary, commonly used as a proxy measure of listening comprehension in the PC literature, was measured using the WIAT-3 receptive vocabulary subtest, during which participants indicate which of four pictures best illustrates a word they hear (Pearson, 2009). Lastly, the Wechsler Intelligence Scale for Children–Fifth Edition was used to measure nonverbal IQ, which included block design, matrix reasoning, coding, figure weights, visual puzzles, and picture span subtests, which were summed and standardized to create the nonverbal index score (Wechsler, 2014). Raw behavioral assessment scores, along with demographic information including age and sex, were then used to determine group classification and were used as covariates in analyses (see Table 1).

**Table 1.** Demographic information for all participants and classification methods.

	*n* (female)	Age^a^	Reading comprehension^b^	Decoding^b^	Oral vocabulary^b^	Nonverbal IQ
*Gray matter analyses*						
Cutoff classification						
Poor comprehenders	58 (13)	11.57 (2.04)	85.50 (6.00)	106.43 (6.23)	95.83 (17.81)	90.00 (11.76)
Poor decoders	58 (16)	10.82 (1.78)	108.64 (10.24)	82.41 (6.24)	113.22 (9.12)	97.93 (12.12)
Typical readers	58 (13)	11.38 (2.05)	109.45 (10.48)	110.22 (8.23)	114.48 (9.38)	92.66 (10.42)
Mixed regression						
Unexpected poor comprehenders	254 (81)	11.00 (2.05)	86.37 (9.13)	94.11 (16.82)	104.69 (14.77)	97.28 (14.62)
Expected comprehenders	254 (99)	11.15 (2.24)	101.67 (9.18)	99.41 (16.46)	108.50 (14.38)	102.81 (15.85)
Unexpected good comprehenders	254 (103)	10.95 (2.28)	112.91 (15.41)	92.90 (14.44)	103.53 (13.51)	97.16 (14.16)
Regression						
Full sample	1270 (480)	10.99 (2.16)	100.73 (14.76)	96.86 (16.34)	105.66 (15.02)	100.01 (15.12)

*White matter analyses*						
Cutoff classification						
Poor comprehenders	26 (7)	11.60 (2.12)	85.27 (4.80)	106.88 (5.39)	96.62 (18.51)	88.27 (11.29)
Poor decoders	26 (7)	11.12 (1.73)	106.81 (8.47)	80.62 (7.58)	110.42 (7.30)	92.35 (11.87)
Typical readers	26 (7)	11.53 (2.21)	111.42 (12.56)	111.46 (8.59)	112.50 (9.48)	89.15 (10.62)
Mixed regression						
Unexpected poor comprehenders	181 (63)	10.77 (1.97)	86.39 (9.85)	92.41 (17.98)	103.81 (14.51)	96.42 (15.61)
Expected comprehenders	181 (69)	11.02 (2.21)	103.31 (10.60)	99.30 (16.94)	107.50 (14.63)	103.12 (15.57)
Unexpected good comprehenders	181 (65)	11.02 (2.29)	113.52 (15.54)	92.65 (13.94)	103.49 (13.79)	95.89 (13.96)
Regression						
Full sample	903 (336)	11.05 (2.18)	102.11 (15.58)	96.83 (16.44)	105.57 (14.85)	100.08 (15.35)

^a^ In all relevant cases, mean is reported with standard deviation in parenthesis.

^b^ All behavioral scores reported are standardized scores for interpretability, while raw scores were used in all models. All behavioral tests have a test mean of 100 and a standard deviation of 15.

### Group Classification

**Figure 2.**
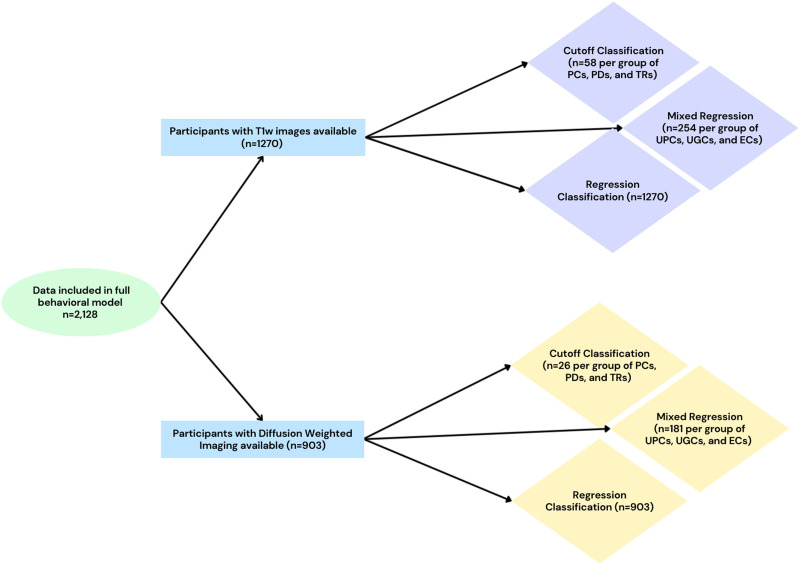
A graphical representation of the data selection and group classification processes used in the current analyses. PCs = poor comprehenders; PDs = poor decoders; TRs = typical readers; UPCs = unexpected poor comprehenders; UGCs = unexpected good comprehenders; ECs = expected comprehenders.

Three widely used classification approaches were utilized for assessing PCs: (a) a cutoff classification model, which identifies PCs based on standard scores indicating poor reading comprehension and good decoding and compares them to age-, sex-, and nonverbal IQ-matched poor decoders and good readers; (b) a mixed classification model, which uses regression to predict reading comprehension from decoding, vocabulary, age, sex, and nonverbal IQ, and then separates the residuals from that regression model into quintiles, with the tails forming the unexpected PC and unexpected good comprehender groups and the middle quintile forming the expected comprehender groups; and (c) a continuous approach, which jettisons groups altogether and uses the regression residuals from (b) to predict the brain measures (see Figure 2 for a graphical representation of these classification methods).

#### Cutoff classification model

This model creates cutoff-based groups of PCs, poor decoders, and typical readers. Specifically, we identified PCs (*n* = 58 for gray matter analysis, *n* = 26 for white matter analysis) as those with typical decoding (decoding standard score of ≥100) and lower than average reading comprehension (reading comprehension standard score of ≤90). We then identified age-, sex-, and nonverbal IQ-matched groups of poor decoders (*n* = 58 gray matter, *n* = 26 white matter, who had decoding standard scores of ≤90 and reading and oral language standard scores of ≥100) and typical readers (*n* = 58 gray matter, *n* = 26 white matter who had decoding, reading, and oral language standard scores of ≥100) using the *MatchIt!* R package (Version 4.5.5, optimal method, Mahlanobis distance) (Ho et al., 2011). Gray and white matter groups were matched independently. One outlier was identified in the PC gray matter group, with a reading comprehension score that was 10 points lower than the next poorest comprehending peer, though no other behavioral or brain measures were found to be significantly different from the group. All models were run with and without the outlier to assess possible skew in results.

#### Mixed classification model

This model uses regression to predict reading comprehension from decoding, oral language abilities, and control variables (age, sex, and nonverbal IQ). All participants with behavioral data (*n* = 2,129) were included in this model to provide the most representative distribution of reading scores available given our inclusion criteria. After modeling the relationship between reading comprehension and other variables, residual scores were used to define group membership, and the lowest, middle, and highest 20% of residual scores with imaging data available were retained for analysis (Li & Kirby, 2014). Participants with residual scores in the 40th to 60th percentiles (which were generally close to zero, indicating little difference between expected and measured reading comprehension) formed the “expected comprehender group” (*n* = 254 gray matter, *n* = 181 white matter). Those with positive residual scores in the 80th to 100th percentiles (indicating better than predicted reading comprehension) formed the “unexpected good comprehender group” (*n* = 254 gray matter, *n* = 181 white matter). Participants with negative residual scores in the 0th to 20th percentiles (indicating worse than predicted reading comprehension) formed the “unexpected PC group” (*n* = 254 gray matter, *n* = 181 white matter). No outliers were identified in these groups.

#### Regression classification model

In this final model, residual scores from the mixed classification model detailed above were directly used to predict brain measures (*n* = 1,270 gray matter, *n* = 903 white matter). See Table 1 for a description of participants across all classification models.

### Statistical Analysis

Across both gray and white matter, linear models were estimated for each classification approach and for each brain metric across the whole brain. Age, nonverbal IQ scores, and imaging quality measures (CNR for both gray and white matter) were centered in all analyses. All models used group status (PC, typical readers, etc.) or residual score and age, sex, nonverbal IQ, and MRI quality control measures as covariates to estimate brain structure. In models using group status, group was dummy coded with PCs being coded as 1 in all cases. All models were fit in R (Version 4.3.2) using the *lm* function and corrected for multiple comparisons using the Benjamini–Hochberg false discovery rate correction (Benjamini & Hochberg, 1995; R Core Team, 2023). Corrections were applied to each dependent variable of interest independently for each classification method across both gray and white matter analyses for each contrast (PCs compared to poor decoders, typical readers, and all other readers combined, etc.).

After comparison of outputs for our group analyses and regression analyses and visual inspection of the data for all regions, we suspected potential nonlinear relationships between our independent and dependent variables (regression residuals, brain structure) for some tracts of interest and thus also ran the regression models with the inclusion of a quadratic term. Again all models were fit in R (Version 4.3.2) using the *lm* function, and model fits were compared to linear model fits using ANOVA. The ANOVA results were corrected for multiple comparisons using the Benjamini–Hochberg false discovery rate correction.

## RESULTS

### White Matter

#### Cutoff classification method

No significant differences in white matter structure (FA, MD, NDI, ODI) were identified by the cutoff classification models.

#### Mixed regression classification method

**Figure 3.**
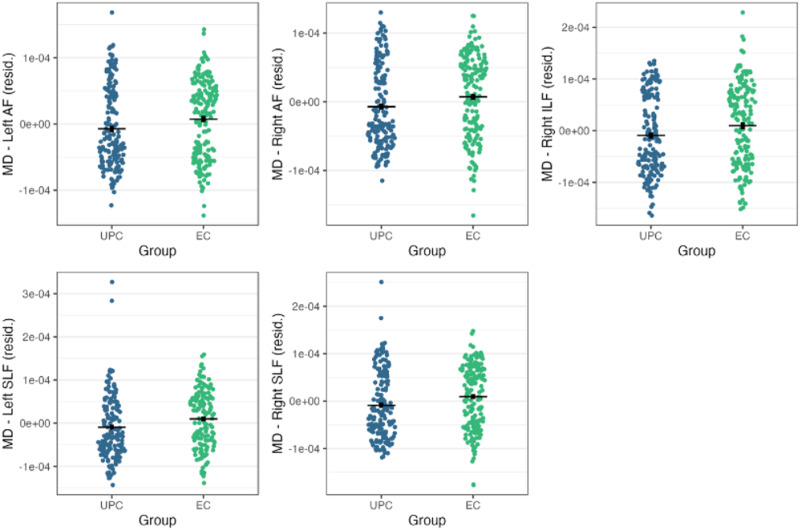
Scatter plots of significant hypothesized group differences in mean diffusivity (MD) from the contrast with the largest effect size. The horizontal line represents the group mean while the thick black vertical lines represent ±1 *SE*. UPC = unexpected poor comprehenders; EC = expected comprehenders. Cook’s distance was used to evaluate influential outliers, and all data points had a *D* > 0.5.

In the mixed regression classification method, small but systematic differences in white matter structure were found between unexpected PCs and other readers. Specifically, unexpected PCs showed increased MD as compared to both expected comprehenders and unexpected good comprehenders across a number of left and right hemisphere tracts, including hypothesized tracts, with small to medium effect sizes (all results *p* < 0.05 after FDR correction, see Table 2 for a full list of tracts that differed between groups, including standardized beta estimates and effect sizes). These differences were found in tracts that have commonly been linked to variation in reading and language, including hypothesized differences in the bilateral SLF, ILF, and AF, and additional differences in the bilateral inferior fronto-occipital fasciculus, and middle longitudinal fasciculus (see Figure 3 for scatter plots associated with hypothesized effects). Increased MD for unexpected PCs as compared to expected comprehenders and unexpected good comprehenders was also found in tracts that have been correlated with primarily domain-general processes (i.e., attention), including the anterior commissure, bilateral fornix, bilateral uncinate fasciculus, and others.

**Table 2.**
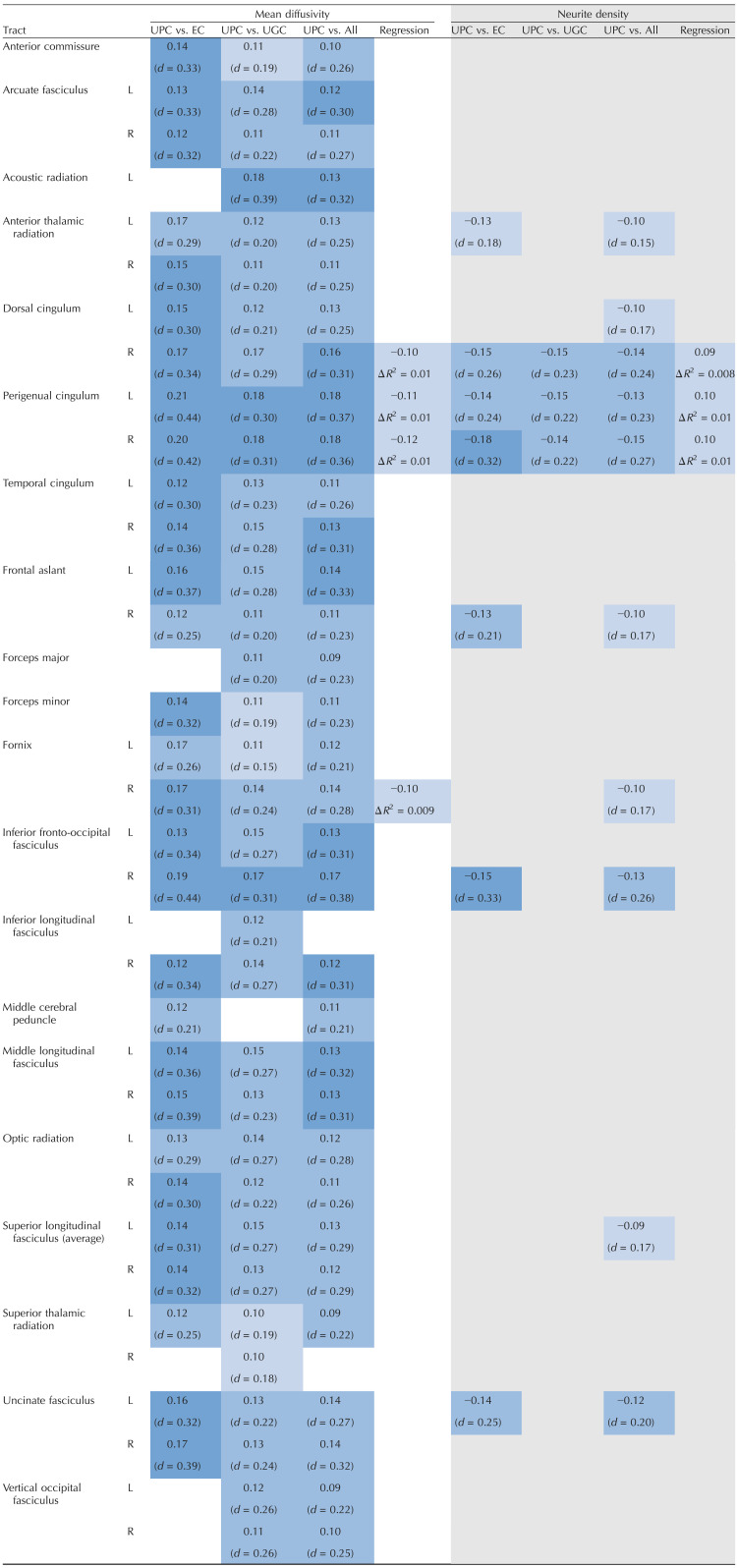
All white matter results that remained significant (*p* < 0.05) after Benjamini–Hochberg false discovery rate multiple-comparisons correction. Standardized beta estimates and effect sizes or Δ*R*^2^ are reported. Results are color-coded by effect size, with smallest effect sizes being colored in the lightest shade of blue and the strongest effect sizes being colored in the darkest shade of blue.

*Note*. UPC = unexpected poor comprehender; EC = expected comprehender; UGC = unexpected good comprehender; All = combined expected comprehender and unexpected good comprehender group; Regression = differences seen in the regression classification; L = left; R = right.

Similarly, unexpected PCs were found to have decreased NDI as compared to both expected comprehenders and unexpected good comprehenders in a more limited number of tracts (see Table 2). Like with MD, some of these differences were found in tracts that connect regions involved with language processing, including the left SLF and right ILF. Additionally, a small number of tracts that have been linked to domain-general processes were implicated including the left anterior thalamic radiation and uncinate, right fornix and temporal cingulum, and bilateral dorsal and perigenual cingulum and frontal aslant.

No statistically significant differences were observed between unexpected PCs and peers in FA or ODI.

#### Regression classification method

In the regression classification, for linear models, participants with more negative residual scores (those whose comprehension was poorer than expected) were found to have increased MD as compared to those with more positive residual scores in the right dorsal cingulum, fornix, and bilateral perigenual cingulum.

Two of these results were mirrored in the NODDI measures, which indicated that those with more negative residual scores had decreased NDI as compared to those with more positive residual scores in the right dorsal cingulate and bilateral perigenual cingulum.

**Figure 4.**
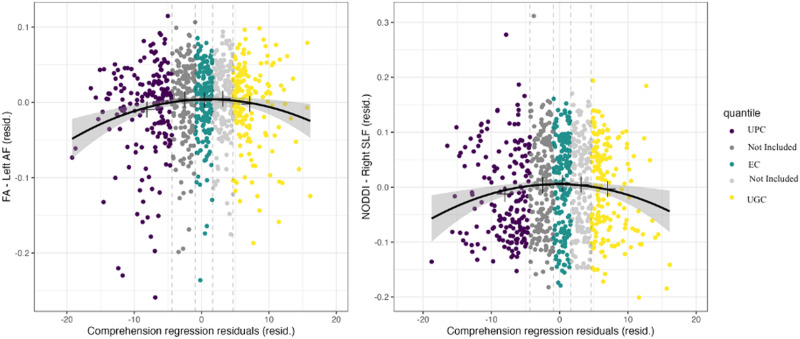
Scatter plots of hypothesized significant quadratic results. All results from the regression classification method. To better visualize how these analyses differed from the group results shown above, data are colored to represent group membership from the mixed regression classification, with purple dots representing unexpected poor comprehenders (UPC), turquoise representing expected comprehenders (EC), yellow representing unexpected good comprehenders (UGC), and gray dots representing participants not included in group contrasts. Group means are indicated by a + symbol.

Outputs from the models that included quadratic terms revealed that reading comprehension was significantly associated with integrity in a number of tracts, including two hypothesized effects that were not identified in the linear models—reduced FA for more unexpected PCs (relative to those with more expected or good comprehension) in the left AF and right ILF for more unexpected PCs (relative to those with more expected or good comprehension). Note that while these models were fit with a quadratic function (Figure 4), the relationship appears to be driven by lower FA for more unexpected poor comprehension (those with negative residuals) vs. everyone else (those with residuals near 0 and positive residuals).

### Gray Matter Analyses

No statistically significant results were observed after correction for multiple comparisons for any classification models. However, we did find a number of nominally significant results, which are consistent with prior literature (see Supporting Information Table 3, for full details of these results).

## DISCUSSION

The current study sought to determine whether PCs differ from typical readers and/or poor decoders in their brain structure. Utilizing a large and diverse sample and multiple classification approaches, the current paper provides a robust whole-brain comparison of cortical morphometry and white matter microstructural integrity in PCs, typical readers, and/or poor decoders.

**Figure 5.**
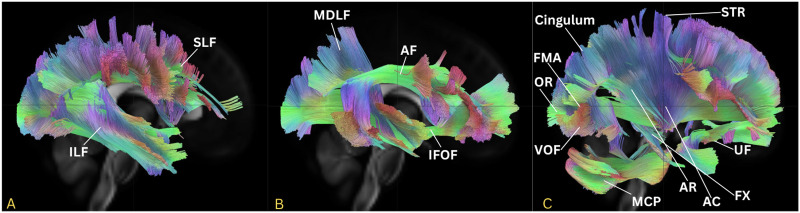
Panel A shows hypothesized white matter tracts with significantly worse integrity in UPCs than other readers. Panel B shows language-related tracts with significantly worse integrity in UPCs than other readers. Panel C shows domain-general tracts with significantly worse integrity in UPCs than other readers. All tract abbreviations can be found in Supporting Information Table 1. UPCs = unexpected poor comprehenders.

Our results indicate widespread but small differences in white matter tract integrity among PCs, poor decoders, and typical readers. Group differences were observed bilaterally in tracts previously linked to language ability as well as in tracts associated with more domain-general processes (see Figure 5). Some of these findings align with prior neuroimaging studies of reading comprehension and were therefore hypothesized, whereas others represent novel observations. Notably, a greater number of statistically significant effects and larger effect sizes were observed for MD and NDI than for FA, suggesting that these metrics may be more sensitive to comprehension-related variation in white matter microstructure, potentially reflecting underlying neurodevelopmental processes such as neuronal migration, axon guidance, myelination, and synaptic pruning.

To focus our discussion on the most robust findings, we limit interpretation to effects observed in hypothesized regions or tracts and to novel findings with effect sizes at or above *d* = 0.30 or Δ*R*^2^ = 0.022, corresponding to a small-to-medium effect size.

### White Matter Analyses

Although no prior studies have examined white matter integrity in PCs specifically, some of our results align with findings from research on individual differences in reading comprehension among typically developing readers and were therefore anticipated (Horowitz-Kraus et al., 2014, 2015). Specifically, in the mixed classification models, unexpected PCs showed reduced white matter integrity—indexed by increased MD and decreased NDI—relative to expected comprehenders and unexpected good comprehenders in the bilateral SLF, ILF, and AF. These effects were small to small-to-medium in magnitude (*d* = 0.17–0.34). In nonlinear regression classification models, we also observed reduced FA in the left AF and in the right ILF for more unexpected PCs (those with more negative residuals) relative to those with more expected comprehension or unexpected good comprehension, and reduced NDI in the SLF for more unexpected PCs (those with more negative residuals) relative to those with more expected comprehension or unexpected good comprehension. While MD revealed a linear relationship, FA only became significant in the quadratic models. This suggests that white matter structural integrity remains stable for most participants but declines sharply in those with very poor reading comprehension as compared to their decoding and oral language abilities. This discrepancy likely reflects MD’s higher sensitivity to general tissue density versus FA’s specificity to the distinct structural disorganization found in unexpectedly PCs. Although several additional language- and reading-related tracts (e.g., left inferior fronto-occipital fasciculus, left middle longitudinal fasciculus; see Table 3) were significantly associated with poor comprehension in some models, (Eze et al., 2024; Koirala et al., 2021; Luo et al., 2020; Shekari & Nozari, 2023; Turken & Dronkers, 2011; Vanderauwera et al., 2018). These tracts have not been previously linked specifically to reading comprehension. As such, they were not hypothesized a priori and did not meet our criteria for focused discussion.

**Table 3.**
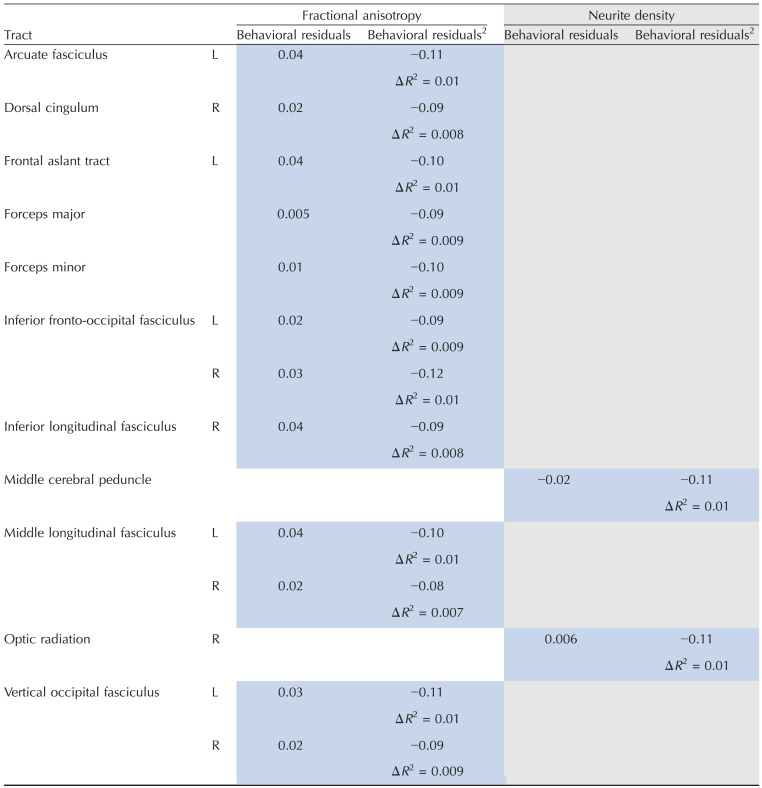
All significant results from nonlinear analysis, conducted in the regression classification models, after correcting for multiple comparisons. Standardized beta estimates for both the standard residuals term and the squared residuals term are presented, along with the change in *R*^2^ values when adding the nonlinear term to the model.

*Note*. L = left; R = right.

We also identified several novel effects that met our threshold for discussion (*d* ≥ 0.30). Many of the implicated tracts have been linked to both language-specific and more general cognitive functions, including the bilateral uncinate fasciculus, left frontal aslant tract, and left acoustic radiation. A recent review suggests that the uncinate fasciculus plays a role in semantic processing, emotional processing, and learning, with evidence from studies of primary progressive aphasia and correlations between tract integrity and semantic task performance (Shekari & Nozari, 2023). Similarly, the frontal aslant tract has been implicated in both language and domain-general cognitive processes and is thought to support language learning, comprehension, and fluency, as well as speech motor control. Reviews have highlighted associations between left frontal aslant tract integrity and stuttering severity, as well as executive functions, particularly inhibitory control (Dick et al., 2019; Shekari & Nozari, 2023). The left acoustic radiation, which connects thalamic and temporal regions and relays auditory information, has traditionally been viewed as primarily sensory; however, emerging evidence suggests a broader role in language and auditory processing (Maffei et al., 2019). In addition, we observed reduced white matter integrity in unexpected PCs relative to expected comprehenders in the right optic radiation, a tract associated with visual processing and visuospatial tasks (Groppo et al., 2014; Yoshimine et al., 2018). Collectively, these findings suggest that poor reading comprehension—particularly when unexpected based on decoding skill—may reflect disruptions not only in canonical language pathways but also in broader networks supporting semantic, executive, auditory, and visual processing. These results are consistent with functional imaging research that reveals large, bilateral networks for reading comprehension that include substantial right hemisphere involvement (Aboud et al., 2016; Cutting et al., 2013; Horowitz-Kraus et al., 2014, 2015; Patael et al., 2018; Ryherd et al., 2018).

The final set of tracts with small to medium effect sizes have been associated with memory and information integration processes. In all cases, we observed reduced integrity in unexpected PCs relative to expected comprehenders, unexpected good comprehenders, and a combined group of expected and unexpected good comprehenders. These tracts include the right fornix, anterior commissure, bilateral cingulum, and forceps minor. The fornix has been shown to predict cognitive impairment and to relate to memory performance in young children (Caldinelli et al., 2017; Fletcher et al., 2013; Korbmacher et al., 2023). The anterior commissure has primarily been associated with integrative functions, particularly interhemispheric information transfer (Çavdar et al., 2021). Similarly, the cingulum is thought to support integrative processes and executive functions, with evidence indicating that reduced cingulum integrity is associated with poorer performance on executive function tasks (Bettcher et al., 2016; Maldonado et al., 2020). Notably, differences in the perigenual cingulum yielded the largest effect sizes across all analyses and were significant in both the mixed regression and regression classification models for both MD and NDI, underscoring the potential importance of this structure for reading comprehension in PCs. Although the specific functional contributions of the forceps minor are less well characterized, this tract has been associated with attentional difficulties and bilingualism, potentially reflecting demands on task switching and cognitive control (Hatton et al., 2014; Lawrence et al., 2013; Liu et al., 2021; Mamiya et al., 2018; Zhang et al., 2022). These findings provide converging evidence that inefficiencies in information integration across multiple cognitive domains may represent a core vulnerability for PCs.

Together, these tracts are thought to support functions that are critical for skilled reading comprehension, including language processing, visual and auditory processing, memory, executive functioning, and interhemispheric integration. Overall, our findings indicate that differences in white matter microstructure among PCs are distributed across a broad, bilateral network of tracts rather than localized to a single pathway. This pattern supports the hypothesis that PCs may exhibit widespread yet subtle differences across multiple reading-related subskills, which may be most effectively characterized neuroanatomically through variation in connective (white matter) architecture.

### Gray Matter Analyses

No gray matter differences survived correction for multiple comparisons. However, several hypothesized effects reached nominal significance prior to correction and are reported for completeness and comparison to prior literature. These nominally significant findings primarily involved reduced cortical surface area and increased cortical thickness in frontal, temporal, and parietal regions previously implicated in reading comprehension (e.g., angular gyrus, precentral gyrus, supramarginal gyrus, and superior temporal regions), with effects generally indicating reduced surface area or increased thickness among poorer-than-expected comprehenders. These findings are detailed in the Supporting Information Table 3.

Differences between our findings and those of prior gray matter studies of PCs—many of which reported a larger number of regions surviving statistical thresholds—may reflect both sample characteristics and analytic approach. Our sample was larger and more diverse than those in earlier studies, and prior work suggests that reading-related covariance in brain structure is more readily detected in relatively homogeneous samples with lower variance in environmental and individual-level factors (Roy et al., 2024). Moreover, replication efforts in brain-wide association studies indicate that effect sizes tend to diminish as sample sizes increase and better approximate true population-level effects, which are likely to be small for complex traits such as reading (Marek et al., 2022).

A second consideration is methodological. Our whole-brain analytic approach, which examined approximately 148 regions per metric, required conservative correction for multiple comparisons. Had we instead adopted a region-of-interest approach restricted to areas identified in prior gray matter studies of PCs, several regions noted here would have reached statistical significance (see Supporting Information Table 3). Accordingly, although the nominal findings reported should be interpreted cautiously, their convergence with prior work lends support to reading comprehension-related associations in the left inferior parietal lobule and supramarginal gyri, right superior frontal and temporal gyri, and bilateral precentral gyri.

### Classification Comparison

A secondary aim of this study was to compare results across classification approaches. Although expected behavioral patterns were generally consistent across methods, differences emerged in how distinctly reader profiles were defined. The cutoff approach maximized separation between PCs and poor decoders but resulted in smaller group sizes and reduced statistical power. In contrast, the mixed classification approach produced relatively distinct reader groups while retaining larger sample sizes. The regression approach treated reading comprehension as a continuous residualized measure, retained all participants, and did not impose categorical distinctions, thus yielding the largest sample size but losing distinctive groups (see Supporting Information Table 6). These methodological differences likely contributed to variation in brain–behavior findings across approaches.

In the white matter analyses, more effects survived FDR correction in the mixed classification models than in the regression models, and no tracts reached significance in the cutoff models. Notably, all tracts that were significant in the regression models were also significant in the mixed classification models. This pattern is consistent with differences in statistical power: The cutoff approach sacrifices power due to smaller sample sizes, whereas the mixed classification approach balances sample size with contrast strength. Although the regression approach benefits from the largest sample size, retaining intermediate cases may attenuate effects. Excluding these intermediate cases in the mixed classification approach (approximately 20%) may therefore contribute to more significant findings. Overall, effect patterns were broadly similar across approaches, particularly between the two residual-based methods.

### Conclusion

In conclusion, our findings suggest that reading comprehension-specific difficulties are associated with a diffuse, bilateral network of brain regions and white matter tracts implicated in language processing, memory, emotion, and executive function. The white matter findings were robust across analytic approaches, whereas gray matter effects were more limited and did not survive correction for multiple comparisons and thus should be interpreted cautiously. This overall neurobiological pattern is consistent with the broad yet subtle behavioral difficulties observed in PCs, particularly for integrative processes such as comprehension monitoring and inference making. Using a large and diverse sample, our results partially replicate prior gray matter findings in PCs and extend this work by identifying complementary white matter associations. Together, these findings support multifactorial models of poor comprehension that extend beyond the Simple View of Reading to include domain-general processes that may be important for reading comprehension but less central to listening comprehension or word decoding. Future work should build on these results by examining functional activation during reading comprehension in similarly large and diverse samples.

## ACKNOWLEDGMENTS

The authors would like to acknowledge the intellectual contributions of Dr. Fumiko Hoeft and Dr. Sumarga H. Suanda, who provided invaluable feedback on this project throughout its development.

## FUNDING INFORMATION

Kelly Mahaffy, National Institute on Deafness and Other Communication Disorders (https://dx.doi.org/10.13039/100000055), Award ID: T32 DC017703.

## AUTHOR CONTRIBUTIONS

**Kelly Mahaffy**: Conceptualization; Data curation; Formal analysis; Methodology; Software; Visualization; Writing – original draft. **Nabin Koirala**: Data curation; Methodology; Software; Writing – review & editing. **Daniel Kleinman**: Data curation; Formal analysis; Methodology; Software; Validation; Visualization; Writing – review & editing. **Nicole Landi**: Conceptualization; Project administration; Resources; Supervision; Writing – review & editing.

## DATA AVAILABILITY STATEMENT

All data and code used for this project, along with project preregistration, are publicly available in an OSF repository: https://osf.io/2ng57/overview.

## Note

^1^ Note that in our imaging samples (those with gray matter or white matter data), there were eight confirmed cases of DLD, with six of those eight participants not included in a group contrast for any analyses and one participant in each of the unexpected PC and unexpected good comprehender groups.

## Supplementary Material



## TECHNICAL TERMS

Poor comprehenders: People with poor reading comprehension ability despite having typical word reading skill and IQ.
Poor decoders: People who struggle to read new or unfamiliar words or map sounds to letters.
Unexpected poor comprehenders: People whose reading comprehension is poorer than predicted based on their decoding and oral language skills.
White matter: Protected connective tissue in the brain that can increase processing efficiency.
Fractional anisotropy: A measure of the shape of diffusion of water around a white matter tract.
Mean diffusivity: A measure of the amount of water that diffuses across a particular white matter tract.
Neurite density index: An estimate of the number of neurons within a given white matter tract.
Unexpected good comprehenders: People whose reading comprehension is better than predicted based on their decoding and oral language skills.
Expected comprehenders: People whose reading comprehension is similar to what is predicted based on their decoding and oral language skills.
