# Supplementary material for: Structural Brain Correlates of Poor Reading Comprehension
Source: Neurobiol Lang (Camb). 2026 Jul 1;7:NOL.a.265. doi: 10.1162/NOL.a.265 (PMC13379302; doi:10.1162/NOL.a.265)
Supplement: Supplementary file 1 [file nol-07-265-s001.docx]

**Supplementary Material**

**Structural brain correlates of poor reading comprehension**

Kelly Mahaffy,^1,3^ Nabin Koirala,^2,3,4^ Daniel Kleinman^3^ and Nicole Landi^1,2,3^

**White matter tracts and abbreviations**

**Supplemental Table 1**: **White matter tracts and abbreviations**

| **Tract** | **Abbreviation** |
| --- | --- |
| Anterior Commissure | AC |
| Arcuate Fasciculus (bilateral) | Left AF, Right AF |
| Acoustic Radiation (bilateral) | Left AR, Right AR |
| Anterior Thalamic Radiation (bilateral) | Left ATR, Right ATR |
| Dorsal Cingulum (bilateral) | Left CBD, Right CBD |
| Perigenual Cingulum (bilateral) | Left CBP, Right CBP |
| Temporal Cingulum (bilateral) | Left CBT, Right CBT |
| Corticospinal Tract (bilateral) | Left CST, Right CST |
| Frontal Aslant Tract | Left FAT, Right FAT |
| Forceps Major | FMA |
| Forceps Minor | FMI |
| Fornix (bilateral) | Left FX, Right FX |
| Inferior Longitudinal Fasciculus (bilateral) | Left ILF, Right ILF |
| Inferior Fronto-Occipital Fasciculus (bilateral) | Left IFOF, Right IFOF |
| Middle Cerebellar Peduncle | MCP |
| Middle Longitudinal Fasciculus (bilateral) | Left MDLF, Right MDLF |
| Optic Radiation (bilateral) | Left OR, Right OR |
| Superior Longitudinal Fasciculus (bilateral)^a^ | Left SLF, Right SLF |
| Superior Thalamic Radiation (bilateral) | Left STR, Right STR |
| Uncinate Fasciculus (bilateral) | Left UF, Right UF |
| Vertical Occipital Fasciculus (bilateral) | Left VOF, Right VOF |

^a^In this study, the three segments of the SLF were averaged in each hemisphere to create a single SLF variable per measure used for all analyses.

**Age Distribution of Participants**

This study included a large age range of participants (8-16 years). The age distribution was left skewed, with more younger participants than older participants.

**Supplemental Figure 1: Age Distribution of the Imaging Sample**


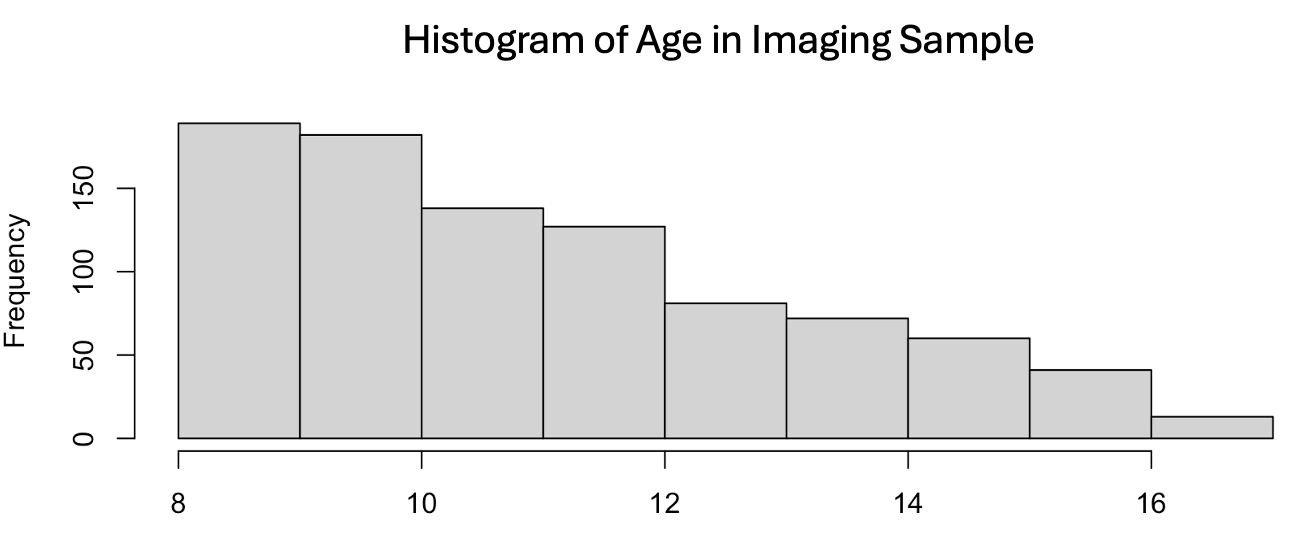
**Supplemental Figure 1:** A histogram of the age distribution of participants from our imaging sample, including 1,270 children with T1w MRI imaging data available. All 903 children with white matter data available also had T1w data available.

**Participants excluded for disorders**

In this study, participants with a predefined set of diagnoses and disorders were excluded from all analyses so as to not add additional variability in brain structure likely due to a comorbidity. In total, 228 children who met other inclusion criteria were excluded because of a diagnosis.

**Supplemental Table 2: Participants removed from analyses due to clinician confirmed diagnoses.**

| **Clinician Confirmed or Suspected Diagnosis** | **Number of Participants Removed** |
| --- | --- |
| Autism Spectrum Disorder | 170 |
| Borderline Intellectual Functioning | 19 |
| Speech Sound Disorder | 15 |
| Pragmatic Communication Disorder | 10 |
| Intellectual Disability- Mild | 10 |
| Selective Mutism | 6 |
| Fetal Alcohol Related Neurodevelopmental Delay | 1 |
| *Total Participants Removed for Diagnosis* | *228* |

**Untimed Reading Scores by Group**

In addition to speeded word reading assessments (the TOWRE), all participants completed untimed word and pseudo-word reading subtests from the Weschler Individual Achievement Test (WIAT)-III. In these assessments, children read real words or pronounceable pseudowords until they make four consecutive errors. Standard scores from those assessments are reported by group. Both subtests have a mean of 100 and a standard deviation of 15.

**Supplemental Table 3: Untimed Reading Scores by Group**

|  | Word Reading | Pseudoword Reading |
| --- | --- | --- |
| *Grey Matter Analyses* |  |  |
| **Cutoff Classification** |  |  |
| Poor Comprehenders | 102.73 | 104.45 |
| Poor Decoders | 94.96 | 88.83 |
| Typical Readers | 112.29 | 108.73 |
| **Mixed Regression** |  |  |
| Unexpected Poor Comprehenders | 95.07 | 93.71 |
| Unexpected Good Comprehenders | 102.43 | 97.39 |
| Expected Comprehenders | 105.58 | 101.36 |
| **Regression** |  |  |
| Full Sample | 102.27 | 98.53 |
| *White Matter Analyses* |  |  |
| **Cutoff Classification** |  |  |
| Poor Comprehenders | 102.19 | 104.08 |
| Poor Decoders | 94.12 | 85.12 |
| Typical Readers | 111.96 | 107.58 |
| **Mixed Regression** |  |  |
| Unexpected Poor Comprehenders | 95.56 | 93.87 |
| Unexpected Good Comprehenders | 102.43 | 97.22 |
| Expected Comprehenders | 105.64 | 101.60 |
| **Regression** |  |  |
| Full Sample | 102.99 | 98.93 |

**Comparisons run for all imaging models**

In the current study, the following comparisons were conducted for both grey and white matter analyses: in the cutoff classification model, poor comprehenders (binary coded as 1) were compared to poor decoders, typical readers, and poor decoders and typical readers combined (all binary coded as 0); in the mixed regression classification, unexpected poor comprehenders (binary coded as 1) were compared to unexpected good comprehenders, expected comprehenders, and unexpected good comprehenders and expected comprehenders combined (all binary coded as 0); in the regression classification, residuals were used to predict changes in brain structure continuously with unexpected poor comprehenders having lower residual values than expected comprehending or unexpected good comprehending peers.

**Supplementary grey matter results**

In this study, there were no grey matter results which survived multiple comparisons correction. This is likely due to the large number of comparisons (74 per hemisphere) in our analyses. There were, however, a number of nominally significant findings of note. Poor comprehenders were coded as 1 in all group analyses and had more negative residual scores in regression classification models.

Regions hypothesized to be associated with comprehension skill are italicized.

**Supplemental Table 4: Nominally significant grey matter results**

|  |  | Standardized Beta Estimate | T-Value | | Uncorrected P-Value | | | | |  |
| --- | --- | --- | --- | --- | --- | --- | --- | --- | --- | --- |
| **Grey Matter Volume** | | | | | | | | |  |  |
| **Poor Comprehenders > Typically Reading Peers** | |  |  | |  | | | | |  |
|  | Left Caudate | -0.21 | -2.307 | | 0.023 | | | | |  |
|  | Right Amygdala^c^ | -0.19 | -2.323 | | 0.022 | | | | |  |
|  | Right Caudate | -0.19 | -2.128 | | 0.036 | | | | |  |
|  | Right Cerebellum (White Matter Volume) | -0.21 | -2.349 | | 0.021 | | | | |  |
| **Poor Comprehenders > All other readers** | |  |  | |  | | | | |  |
|  | Right Amygdala^c^ | -0.15 | -2.171 | | 0.031 | | | | |  |
| **Unexpected Poor Comprehenders > Unexpected Good Comprehenders** | | | | | | |  | |  |  |
|  | Right Accumbens | -0.09 | -2.241 | | 0.026 | | | | |  |
|  | Right Cerebellum | -0.08 | -2.071 | | 0.039 | | | | |  |
| **Unexpected Poor Comprehenders > Expected Comprehenders** | | | |  | |  | |  |  |  |
|  | Optic Chiasm | -0.10 | -2.166 | | 0.031 | | | | |  |
| **Cortical Surface Area** | | | | | | | | | | |
| **Poor Comprehenders vs Poor Decoders** | |  |  | |  | | | | |  |
|  | Right Precuneus Gyrus | 0.19 | 2.207 | | 0.045 | | | | |  |
|  | Right Anterior Collateral Sulcus | 0.20 | 2.481 | | 0.015 | | | | |  |
|  | Right Subparietal Sulcus | 0.18 | 2.030 | | 0.045 | | | | |  |
| **Poor Comprehenders vs Typical Readers** | |  |  | |  | | | | |  |
|  | Left Orbital Gyrus^a^ | -0.18 | -2.073 | | 0.041 | | | | |  |
|  | Left Rectus Gyrus | -0.20 | -2.250 | | 0.026 | | | | |  |
|  | Left Olfactory Sulcus | -0.16 | -2.071 | | 0.041 | | | | |  |
|  | Right Paracentral Gyrus and Sulcus | -0.22 | -2.441 | | 0.016 | | | | |  |
|  | Right Orbital Gyrus^a^ | -0.19 | -2.038 | | 0.044 | | | | |  |
|  | Right Middle Posterior Cingulate^b^ | -0.18 | -2.038 | | 0.044 | | | | |  |
| **Poor Comprehenders vs All Other Readers** | |  |  | |  | | | | |  |
|  | Left Orbital Gyrus^a^ | -0.15 | -1.979 | | 0.050 | | | | |  |
|  | Right Paracentral Gyrus and Sulcus | -0.164 | -2.152 | | 0.033 | | | | |  |
| **Unexpected Poor Comprehender vs Unexpected Good Comprehender** | | | | | |  | | | | |
|  | *Left Angular Gyrus*^c^ | -0.09 | -2.029 | | 0.043 | | | | |  |
|  | *Left Supramarginal Gyrus* | -0.09 | -2.086 | | 0.037 | | | | |  |
|  | Left Superior Parietal Gyrus | -0.09 | -2.197 | | 0.028 | | | | |  |
|  | *Left Precentral Gyrus* | -0.10 | -2.446 | | 0.015 | | | | |  |
|  | Left Precuneus Gyrus | -0.10 | -2.565 | | 0.011 | | | | |  |
|  | Left Sulcus of Jensen | -0.09 | -2.172 | | 0.031 | | | | |  |
|  | Right Paracentral Gyrus and Sulcus | -0.09 | -2.050 | | 0.041 | | | | |  |
|  | *Right Anterior Cingulate Gyrus and Sulcus^c^* | -0.07 | -2.043 | | 0.042 | | | | |  |
|  | Right Orbital Gyrus | -0.09 | -2.036 | | 0.042 | | | | |  |
|  | Right Central Insula | -0.08 | -2.005 | | 0.046 | | | | |  |
|  | Right Angular Gyrus | -0.10 | -2.276 | | 0.023 | | | | |  |
|  | Right Supramarginal Gyrus^c^ | -0.09 | -2.118 | | 0.035 | | | | |  |
|  | *Right Precentral Gyrus^c^* | -0.12 | -2.720 | | 0.007 | | | | |  |
|  | Right Anterior Insular Sulcus | -0.11 | -2.804 | | 0.005 | | | | |  |
|  | Right Lateral Occipito-temporal Sulcus | -0.08 | -2.016 | | 0.044 | | | | |  |
|  | Right Callosal Sulcus | -0.09 | -2.122 | | 0.035 | | | | |  |
| **Unexpected Poor Comprehenders vs All Other Readers** | | | |  | |  | | | | |
|  | Left Superior Parietal Area | -0.07 | -2.068 | | 0.040 | | | | |  |
|  | Right Orbital Gyrus | -0.08 | -2.268 | | 0.024 | | | | |  |
|  | Right Anterior Insular Sulcus | -0.07 | -2.154 | | 0.032 | | | | |  |
|  | Right Lateral Occipito-temporal Sulcus | -0.07 | -2.176 | | 0.030 | | | | |  |
| **Regression** |  |  |  | |  | | | | |  |
|  | Left Posterior Ventral Cingulate^c^ | 0.06 | 2.099 | | 0.036 | | | | |  |
|  | *Left Angular Gyrus^c^* | 0.06 | 2.075 | | 0.038 | | | | |  |
|  | *Left Planum Temporale* | 0.06 | 2.067 | | 0.039 | | | | |  |
|  | Left Central Sulcus | 0.06 | 2.158 | | 0.031 | | | | |  |
|  | Left Sulcus of Jensen | 0.05 | 1.962 | | 0.050 | | | | |  |
|  | Right Paracentral Gyrus and Sulcus | 0.06 | 2.293 | | 0.022 | | | | |  |
|  | *Right Precentral Gyrus^c^* | 0.06 | 2.016 | | 0.044 | | | | |  |
| **Cortical Thickness** | | | | | | | | | | |
| **Poor Comprehenders vs Poor Decoders** | |  |  | |  | | | | |  |
|  | *Left Supramarginal Gyrus* | 0.16 | 2.120 | | 0.036 | | | | |  |
|  | Left Postcentral Gyrus | 0.19 | 2.033 | | 0.044 | | | | |  |
|  | Left Orbital Sulcus | -0.20 | -2.117 | | 0.037 | | | | |  |
|  | *Right Superior Planum Polare Gyrus* | 0.17 | 2.154 | | 0.033 | | | | |  |
|  | Right Vertical Lateral Fissure | -0.23 | -2.288 | | 0.024 | | | | |  |
|  | Right Circular Sulcus (of the Insula) | -0.23 | -2.476 | | 0.015 | | | | |  |
|  | Right Collateral Sulcus^b^ | 0.20 | 2.020 | | 0.046 | | | | |  |
| **Poor Comprehenders vs Typical Readers** | |  |  | |  | | | | |  |
|  | Left Anterior Insular Sulcus^b^ | 0.18 | 2.021 | | 0.046 | | | | |  |
|  | Left Temporal Transverse Sulcus | 0.20 | 2.132 | | 0.035 | | | | |  |
|  | Right Rectus Gyrus | 0.24 | 2.604 | | 0.011 | | | | |  |
|  | Right Pericallosal Sulcus | 0.18 | 2.033 | | 0.045 | | | | |  |
| **Poor Comprehenders vs All Other Readers** | |  |  | |  | | | | |  |
|  | Left Olfactory Sulcus^b^ | 0.15 | 2.020 | | 0.045 | | | | |  |
|  | *Left Temporal Transverse Sulcus* | 0.19 | 2.374 | | 0.019 | | | | |  |
|  | Right Rectus Gyrus | 0.19 | 2.406 | | 0.017 | | | | |  |
|  | *Right Superior Planum Polare Gyrus* | 0.13 | 2.220 | | 0.028 | | | | |  |
|  | Right Olfactory Sulcus | 0.19 | 2.500 | | 0.013 | | | | |  |
| **Unexpected Poor Comprehenders vs Unexpected Good Comprehenders** | | | |  | |  | | | | |
|  | *Left Supramarginal Gyrus* | 0.07 | 2.153 | | 0.032 | | | | |  |
|  | Left Middle Frontal Sulcus^c^ | 0.09 | 2.127 | | 0.034 | | | | |  |
|  | Right Posterior Cingulate Cortex | -0.10 | -2.340 | | 0.020 | | | | |  |
|  | Right Lateral Orbital Sulcus | 0.09 | 2.055 | | 0.040 | | | | |  |
| **Unexpected Poor Comprehenders vs Expected Comprehenders** | | | |  | |  | | | | |
|  | Left Circular Sulcus | 0.10 | 2.371 | | 0.018 | | | | |  |
|  | *Right Fronto-marginal Gyrus and Sulcus* | 0.12 | 2.754 | | 0.006 | | | | |  |
|  | Right Cuneus Gyrus | 0.09 | 2.199 | | 0.028 | | | | |  |
|  | *Right Superior Frontal Sulcus* | 0.09 | 2.100 | | 0.036 | | | | |  |
|  | Right Orbital Sulcus | 0.09 | 2.067 | | 0.039 | | | | |  |
| **Unexpected Poor Comprehenders vs All Other Readers** | | | |  | |  | | | | |
|  | Left Middle Frontal Sulcus^c^ | 0.08 | 2.256 | | 0.024 | | | | |  |
|  | Right Ventral Posterior Cingulate Gyrus | -0.09 | -2.488 | | 0.013 | | | | |  |
|  | Right Orbito-frontal Sulcus | 0.08 | 2.183 | | 0.029 | | | | |  |
| **Regression** |  |  |  | |  | | | | |  |
|  | Left Middle Temporal Gyrus | -0.05 | -2.088 | | 0.037 | | | | |  |
|  | Right Ventral Posterior Cingulate Gyrus | 0.07 | 2.399 | | 0.017 | | | | |  |
|  | Right Circular Sulcus (of the Insula) | -0.05 | -1.972 | | 0.049 | | | | |  |

^a^Result only present without outlier in analysis

^b^Result only present with outlier in analysis

^c^Result mirrors an area implicated in a functional or structural MRI study of PCs, regardless of direction of the finding.

**T-Tests comparing groups by classification method**

One goal of the current study was to compare brain and behavior findings across three common classification methods for poor comprehenders. Groups differed in terms of their behavior, with the cutoff classification producing more distinct groups than the missed regression and regression classification methods. These t-tests compare the poor comprehender, poor decoder, and typical reader groups on decoding, oral vocabulary, and reading comprehension.

**Supplementary Table 5: T-Tests comparing decoding, oral vocabulary, and reading comprehension across groups.**

| **Comparison by Measure** | **T-value** | **p-value** | |
| --- | --- | --- | --- |
| **Decoding** | | |  |
| Poor Comprehenders vs. Typical Readers | 1.774 | 0.078 | |
| *Poor Comprehenders vs. Poor Decoders* | -17.884 | <0.001 | |
| *Poor Decoders vs. Typical Readers* | 18.512 | <0.001 | |
| *Unexpected Poor Comprehenders vs. Expected Comprehenders* | 3.6203 | <0.001 | |
| Unexpected Poor Comprehenders vs. Unexpected Good Comprehenders | 0.354 | 0.724 | |
| *Unexpected Good Comprehenders vs. Expected Comprehenders* | -3.588 | <0.001 | |
| **Oral Vocabulary** |  |  | |
| *Poor Comprehenders vs. Typical Readers* | 5.765 | *<0.001* | |
| *Poor Comprehenders vs. Poor Decoders* | 4.819 | <0.001 | |
| *Poor Decoders vs. Typical Readers* | 1.612 | 0.120 | |
| *Unexpected Poor Comprehenders vs. Expected Comprehenders* | 2.404 | 0.017 | |
| Unexpected Poor Comprehenders vs. Unexpected Good Comprehenders | 0.342 | 0.732 | |
| *Unexpected Good Comprehenders vs. Expected Comprehenders* | -2.103 | 0.036 | |
| **Reading Comprehension** |  |  | |
| *Poor Comprehenders vs. Typical Readers* | 17.464 | <0.001 | |
| *Poor Comprehenders vs. Poor Decoders* | 17.296 | <0.001 | |
| Poor Decoders vs. Typical Readers | 1.015 | 0.312 | |
| *Unexpected Poor Comprehenders vs. Expected Comprehenders* | 15.704 | <0.001 | |
| *Unexpected Poor Comprehenders vs. Unexpected Good Comprehenders* | 22.319 | <0.001 | |
| *Unexpected Good Comprehenders vs. Expected Comprehenders* | 6.660 | <0.001 | |

Italicized contrasts were significantly different across groups.
